# The “Grey Zone” in Blood Donor Screening: A Retrospective Study and Proposal for Donor Re-Entry

**DOI:** 10.3390/diagnostics15172261

**Published:** 2025-09-07

**Authors:** Wajnat A. Tounsi, Nora Y. Hakami, Seraj O. Alamoudi, Wejdan A. Altayeb, Shahad H. Aljuhani, Afnan J. Al-Sulami, Osama A. Alzahrani, Raed M. Garout, Taghreed S. Almansouri, Waleed M. Bawazir, Aisha Qattan, Maha A. Badawi

**Affiliations:** 1 Department of Medical Laboratory Sciences, Faculty of Applied Medical Sciences, King Abdulaziz University, Jeddah 21589, Saudi Arabia; 2 Laboratory Department, King Abdulaziz University Hospital, Jeddah 21589, Saudi Arabia; 3 Blood Transfusion Services Unit, King Abdulaziz University Hospital, Jeddah 21589, Saudi Arabia; 4 Laboratory Department, King Fahad Armed Forces Hospital, Jeddah 23311, Saudi Arabia; 5 Molecular Diagnostic Laboratory, King Abdulaziz University Hospital, Jeddah 21589, Saudi Arabia; 6 Hematology Department, Faculty of Medicine, King Abdulaziz University, Jeddah 21589, Saudi Arabia; 7 Hematology Research Unit, King Fahd Medical Research Center, King Abdulaziz University, Jeddah 22254, Saudi Arabia

**Keywords:** grey zone, transfusion-transmissible infections, blood donors, re-entry protocol

## Abstract

**Background/Objectives**: Grey zone serologic results in blood donor screening pose challenges for transfusion safety, donor management, and blood supply sustainability. In Saudi Arabia, standardized national protocols for managing grey zone outcomes remain lacking. This study aimed to evaluate the prevalence and follow-up outcomes of grey zone serologic results among blood donors at a Saudi hospital over a five-year period. **Methods**: Serological screening results of six transfusion-transmissible infections (TTIs) markers were extracted alongside nucleic acid testing (NAT) results for HBV, HCV, and HIV. The grey zone was defined as a signal-to-cutoff (S/CO) of 0.90–0.99. Repeat and follow-up results, including subsequent donations, were assessed for seroconversion. **Results**: A total of 48,241 donations from 38,524 donors were analyzed. Anti-HBc showed the highest reactivity (*n* = 2312; 4.8%), followed by HbsAg (*n* = 2292; 0.31%) and syphilis (*n* = 218; 0.5%). Grey zone results were rare, and most frequent in anti-HBc (*n* = 76; 0.16%), HCV (*n* = 39; 0.08%), and HBsAg (*n* = 28; 0.06%). Grey zone-to-reactive conversion upon subsequent donation was rare. Three donors who initially tested in the grey zone for anti-HBc later tested reactive in subsequent donations, but their HBV NAT remained negative. **Conclusions**: While grey zone outcomes were infrequent, a subset involving HBV markers showed low-level reactivity on repeat testing. For other TTIs markers, grey zone results likely reflected assay variability rather than true infection. We propose a six-month temporary deferral with follow-up serologic and NAT testing, allowing conditional re-entry for donors with consistently non-reactive results, supporting both transfusion safety and a more sustainable donor pool.

## 1. Introduction

Ensuring the safety of the blood supply is a cornerstone of transfusion medicine. Screening for transfusion-transmissible infections (TTIs), including hepatitis B virus (HBV), hepatitis C virus (HCV), human immunodeficiency virus (HIV), and others, has significantly reduced the risk of transmission. However, grey zone or equivocal serologic results, where the test signal falls 10% below the reactivity threshold, remain a challenge in clinical interpretation, donor management, and blood unit disposition [1,2]. Modern serologic platforms such as chemiluminescent microparticle immunoassays (CMIA) and enzyme-linked immunosorbent assays (ELISA) are designed for high-throughput sensitivity, but often yield borderline signal-to-cutoff (S/CO) values. These “grey zone” results may reflect assay variability, early infection, or transient nonspecific binding. Dow et al. [3] introduced the concept of biological and technical ‘noise’ in microbiological assays, emphasizing how increasing assay sensitivity can reduce specificity and result in random fluctuations near cutoffs, potentially masking early-stage infections or occult cases if not interpreted cautiously [3].

International studies highlight the diagnostic ambiguity of grey zone results. In India, in a study of over 23,000 donors, 0.41% were in the grey zone; among these, 20.6% were reactive upon duplicate testing, 21.7% were indeterminate, and 57.7% were negative [4]. In contrast, Bhardwaj et al. [5] reported that none of 47 grey zone samples from over 50,000 donors were confirmed positive, suggesting that most grey zone results likely reflect assay noise or false positives rather than true infection risk [5]. Similarly, a Swedish study assessed the prevalence and outcomes of false-reactive results in TTIs screening of over 50,000 blood donors [6]. It found that approximately 0.1% of samples fell within the grey zone, yet none were positive upon follow-up testing, supporting the idea that most grey zone results likely reflect assay variability rather than true infection [6].

Other evidence indicates that some grey zone results, particularly in HBV screening, may reflect early or occult HBV infection (OBI) rather than assay noise [7,8]. Grey zone reactivity for antibody to HBV core antigen (anti-HBc) has been linked to OBI, reinforcing the need for structured follow-up algorithms to distinguish true infection from false positivity [4,9]. In the absence of such protocols, donors with transient or borderline reactivity may be unnecessarily deferred, contributing to donor loss and undermining blood supply sustainability. A recent study has explored the use of predictive algorithms to differentiate between true and false-reactive cases, suggesting new opportunities for individualized donor management strategies [10].

False-reactive or equivocal screening results can significantly impact donors, leading to psychological distress and increased workload for blood services. Studies have shown that donors notified of such results experience stress and confusion, with temporary or permanent deferral often resulting in donor loss or reduced return rates [11]. Donors often experience anxiety, confusion, and stigma when notified of unconfirmed results, and blood services may face increased administrative burdens from handling repeat testing, counseling, and documentation. A survey among European blood establishments reported that most centers lack standardized procedures for managing false-reactive donors and highlighted the critical need for re-entry protocols to reduce unnecessary donor loss while maintaining blood safety [11].

Although international studies report varying frequencies of grey zone results in TTIs screening due to differences in assay platforms, populations, and thresholds, they agree on the diagnostic ambiguity these results pose [5,6,11]. They also advocate for re-entry policies that allow donors who are deemed non-infectious to rejoin the donor pool, minimizing unnecessary deferrals while safeguarding the blood supply [8,12]. These studies emphasize the importance of structured management protocols, including clear donor communication, psychological support, and evidence-based follow-up procedures to maintain donor trust and enable safe reintegration [9].

The World Health Organization has acknowledged the lack of high quality evidence supporting many donor deferral decisions, especially in cases involving borderline or ambiguous screening results. In the absence of definitive data, precautionary deferral is often recommended. However, such conservative approaches may inadvertently result in the exclusion of otherwise eligible donors and place strain on blood supply systems [13]. A recent study developed a machine learning-based predictive model using serologic markers and demographic data to distinguish between true and false HBV infections among initially reactive donors. The model, which achieved high accuracy (AUC = 0.936), found that over half of reactive cases were ultimately false positives [10].

In Saudi Arabia, the Ministry of Health mandated nationwide nucleic acid testing (NAT) screening for HBV, HCV, and HIV by the end of 2008 in response to the need for enhanced blood safety [14]. Although NAT represents a significant advancement in screening sensitivity, it is not infallible; low-level viremia, intermittent viral shedding, or viral variants may evade detection [15]. Grey zone testing, which stratifies weakly reactive serologic results and confirms them with NAT, has been shown to enhance safety by identifying cases that standard screening might miss [2,16,17,18]. Despite the routine use of NAT, Saudi Arabia has yet to implement national protocols for managing equivocal results, and systematic reporting on grey zone outcomes remains limited. Although large-scale studies have examined TTIs prevalence, particularly for HBV markers, none have analyzed the implications of grey zone findings [19,20]. The absence of standardized re-entry protocols contributes to inconsistent practices, potential donor loss, and unclear risk assessment. The lack of standardized re-entry protocols and limited data on the clinical and operational implications of grey zone results in Saudi Arabia underscores an urgent need for institution-level evidence to guide national policy. Despite the widespread implementation of NAT, transfusion services still face uncertainty when managing donors with ambiguous serologic profiles.

This study aimed to address these gaps by comprehensively analyzing reactive and grey zone serologic screening results of all blood donors at King Fahad Armed Forces Hospital (KFAFH), Jeddah, from November 2019 to November 2024. Specifically, we aimed to assess the rate of seroconversion, if any, among donors with grey zone results across six TTIs markers. This study is among the first in Saudi Arabia to systematically evaluate grey zone serologic results and their follow-up outcomes, providing critical evidence to inform donor management strategies, optimize the handling of grey zone cases, and ultimately enhance transfusion safety policies.

## 2. Materials and Method

### 2.1. Study Design and Population

This retrospective study analyzed serologic screening results from blood donors who presented to the KFAFH Blood Bank in Jeddah, Saudi Arabia, between November 2019 and November 2024. This study was approved by the Research Ethical Committee of KFAFH (REC795, 25 May 2025). Donor identifier information was anonymized to ensure confidentiality and compliance with ethical standards.

Collected data included donors’ serologic results for HBsAg, anti-HBc, HCV antibody, HIV antigen/antibody combo, HTLV antibody, and syphilis antibody, covering initial screening, Repeat 1 and 2 tests, as well as NAT results for HIV, HCV, and HBV. Donor demographic information (gender, age, and nationality) and the date of donation were also extracted from the KFAFH laboratory blood banking information system (HEMATOS IIG version 5.6) and compiled into Excel spreadsheets for data analysis. Including the donation date allowed us to track subsequent donation outcomes and identify returning donors. Repeat and follow-up donations were reviewed to assess trends in reactivity and seroconversion.

### 2.2. TTIs Testing

Serologic screening for TTIs was performed using CMIA platforms on the ARCHITECT system (Abbott Laboratories, Abbott Park, IL, USA) and, later in the study, the Alinity i system (Abbott Laboratories, USA), following manufacturer protocols. Initial screening results were interpreted as reactive (S/CO ≥ 1.0), grey zone (0.90 ≤ S/CO < 1.0), or non-reactive (S/CO < 0.90). Repeat testing was conducted for all donors who initially screened reactive (S/CO ≥ 1.0) or grey zone (S/CO 0.90–0.99) across all six infection markers. A result was considered reactive if either Repeat 1 or Repeat 2 yielded a value ≥ 1.0. NAT screening was performed using individual donation-NAT (ID-NAT). NAT testing was performed using Procleix Panther (Grifols, Barcelona, Spain) and Cobas 5800 (Roche, Basel, Switzerland) platforms. Results were reported as either positive or negative.

### 2.3. Statistical Analysis

Donor data were anonymized, cleaned, and harmonized for analysis. Each donor was uniquely identified using a coded Donor ID. For cross-sectional prevalence and association analysis, only the first donation per unique donor was used. However, the full donation history was retained to assess longitudinal outcomes such as seroconversion in repeat or subsequent donations. Initial, repeat, and follow-up test results were classified by reactivity status. Donors with grey zone results were analyzed to determine whether they converted to reactive status either through repeat testing or during later donations.

In addition to serologic outcomes, donor characteristics including gender and frequency of donation were examined. Donors were categorized as first-time or repeat donors based on the number of unique donation dates recorded per donor. They were also stratified by gender to assess potential demographic differences in the prevalence of serologic reactivity. The presence of any reactive result in initial screening for HBsAg, anti-HBc, HCV, HIV, HTLV, or syphilis was used as the outcome variable. Associations between donor type, gender, and seroreactivity were evaluated using the chi-square test of independence. Statistical significance was set at *p* < 0.05. All data processing, statistical analysis, and visualization were performed using Python (version 3.13.6).

## 3. Results

### 3.1. Donor Demographics

A total of 48,241 blood donations were recorded during the study period, corresponding to 38,562 unique donors. Of these donors, 96.71% were male (*n* = 46,652) and 3.29% were female (*n* = 1589). The mean donor age was 33.94 years (SD = 9.36), with a median of 33 years and an age range of 18 to 81 years.

### 3.2. Reactive Donors

All 48,241 donors were screened for five TTIs using six serological markers: HBsAg, anti-HBc, HCV, HIV, HTLV, and syphilis. As summarized in Table 1, the overall initial reactivity varied by marker, with the highest rates observed for anti-HBc (4.8%) and HBsAg (4.8%), followed by syphilis (0.5%), HCV (0.3%), HIV (0.2%), and HTLV (0.2%). The majority of initially reactive cases remained reactive on repeat testing for anti-HBc (97.5%), HBsAg (97.4%), and syphilis (92.2%), while lower repeat reactivity rates were observed for HCV (79.0%), HTLV (71.6%), and HIV (20.4%).

### 3.3. Grey Zone Results and Follow-Up

Among 213 blood donors who initially tested in the grey zone for one or more TTIs markers, 43 (20%) returned for subsequent donation. Of these, only three donors (7%)—all for anti-HBc—later tested reactive in the same marker, while HBV NAT remained negative in all subsequent donations (Table 2). The highest proportions of grey zone reactivity were seen in anti-HBc (0.16%), followed by HCV (0.08%), syphilis (0.07%), HBsAg (0.06%), HTLV (0.04%), and HIV (0.03%). Among grey zone donors, reactivity on repeat was most frequently observed for anti-HBc (31.6%), syphilis (30.6%), and HBsAg (28.6%), while repeat reactivity was lower for HCV (28.2%), HTLV (15%), and HIV (0%). Specifically, three donors who initially tested in the grey zone for anti-HBc later tested reactive in subsequent donations. All three donors consistently tested negative for HBsAg and HBV NAT in all subsequent donations. No additional cases of seroconversion were identified among grey zone donors in further subsequent donations. No NAT positivity was detected among donors initially classified as grey zone for any TTIs marker.

### 3.4. Reactivity Associations with Donor Characteristics

Donors were categorized as first-time or repeat donors and stratified by gender to assess associations with initial serologic reactivity. Among first-time donors (*n* = 33,489), 2039 (6.1%) tested reactive, compared to 280 of 5073 repeat donors (5.5%). A chi-square test showed no statistically significant association between donation frequency and reactivity status (χ^2^ = 2.17, *p* = 0.141). In contrast, gender was significantly associated with serologic reactivity. Among male donors (*n* = 37,227), 2904 (7.8%) tested reactive, while 65 of 1335 female donors (4.9%) were reactive. This difference was statistically significant (χ^2^ = 11.75, *p* < 0.001), indicating that male donors were more likely to test reactive than female donors.

## 4. Discussion

This study provides a comprehensive evaluation of serologic screening outcomes, grey zone results, and follow-up patterns among over 38,000 unique blood donors at a tertiary hospital in Saudi Arabia. This study is one of the first large-scale analyses in the region specifically addressing grey zone serologic outcomes and their follow-up, with important implications for blood safety and donor management.

It is important to emphasize that reactivity detected in repeat testing or in subsequent donations does not necessarily indicate the presence of a true infection. Grey zone and initially reactive results can arise from a range of factors, including assay variability, nonspecific binding, cross-reactive antibodies, or transient immune responses, particularly in highly sensitive screening platforms like CMIA or ELISA. Although our study identified a small number of grey zone donors who later tested reactive, these cases cannot be assumed to reflect definitive seroconversion without confirmatory testing by gold-standard methods (e.g., immunoblot). This calls for cautious interpretation of repeat reactivity and the development of formal donor re-entry and follow-up policies that balance blood safety with minimizing unnecessary donor loss.

In TTIs screening, anti-HBc showed the highest reactivity rate (4.8%) among all TTIs markers, consistent with prior reports from Saudi Arabia, where anti-HBc positivity is common, even in the absence of HbsAg [21,22]. This pattern is consistent with a population undergoing epidemiological transition as a result of the longstanding vaccination programs initiated in Saudi Arabia in the early 1990s [23,24,25]. A large study from Riyadh similarly reported low TTIs prevalence among blood donors, with anti-HBc reactivity being the most common (4%) [26].

In Saudi Arabia, mandatory NAT screening for HBV adds a critical layer of safety by enabling early detection of infection and occult HBV cases. At KFAFH, any donation reactive for HBsAg, anti-HBc, or HBV DNA is discarded in accordance with the Association for the Advancement of Blood and Biotherapies (AABB) guidelines, which require all three markers to be non-reactive before transfusion eligibility [27]. Although anti-HBc positivity often reflects past infection, combining serologic and NAT testing improves donor risk assessment and reduces the likelihood of undetected HBV transmission.

Grey zone results were rare across all six markers, with the highest frequencies observed in anti-HBc (0.16%), HBsAg (0.08%), and syphilis (0.07%). Notably, approximately 25–28% of grey zone donors in these categories were reactive on repeat testing, suggesting that a subset of grey zone results may indicate early, low-level infection, or OBI [3]. Among returning grey zone donors, seroconversion was low. Only three donors —all for anti-HBc—later tested reactive in the same marker, and their HBV NAT remained negative in all subsequent donations (Table 2). While a recent study by Bhardwaj et al. [5] reported that grey zone testing has limited value, with none of their 47 grey zone samples positive on further testing, our findings suggest that grey zone results should not be universally dismissed. Although rare, the detection of early or occult infections in grey zone donors supports the need for structured follow-up, confirmatory testing, and re-entry policies to balance blood safety with minimizing unnecessary donor loss. Our results also underscore that the utility of grey zone testing is not uniform across all TTIs and should be evaluated in light of local epidemiology, assay performance, and follow-up capacity, rather than applying generalized policies.

A notably low proportion of donors who initially tested in the grey zone returned for subsequent donation—only 43 out of 213 (20%)—which may reflect an adverse impact of grey zone results on donor retention. While grey zone reactivity often represents nonspecific or transient findings, the ambiguity surrounding such results can lead to donor confusion, anxiety, and perceived deferral even in the absence of formal exclusion. The absence of standardized national guidance on managing grey zone results in Saudi Arabia may further contribute to inconsistent donor communication and re-entry pathways. This potential loss of otherwise healthy and eligible blood donors represents a missed opportunity to sustain the blood supply. Establishing a structured re-entry protocol can help reduce unnecessary donor loss, restore donor confidence, and support long-term blood supply sustainability.

### Proposal of Donor Re-Entry Protocol

The management of donors with inconclusive serologic results, particularly grey zone or low-level reactive cases with negative NAT, remains a significant gap in transfusion practice in Saudi Arabia. In the absence of nationally standardized re-entry protocols, such donors are often permanently deferred, potentially excluding low-risk individuals and compromising blood supply sustainability without corresponding safety benefits.

Aligned with international practices, our findings support a six-month temporary deferral for donors with grey zone reactivity in HBV markers, especially anti-HBc and HBsAg. This mirrors Chinese protocols, which consider six months sufficient to detect seroconversion during the window period [8,12]. Re-entry is allowed only if follow-up testing confirms that HBsAg, anti-HBc, and HBV DNA are all non-reactive [8]. This approach effectively balances transfusion safety and donor retention by detecting early or occult infections while avoiding unnecessary exclusion. Similarly, evidence from the United Kingdom shows that donors with repeat reactive results for markers such as anti-HCV, anti-HIV, and HBsAg can be safely reinstated following negative confirmatory testing after a six-month deferral [28].

Developing a donor re-entry protocol in Saudi Arabia, tailored to local epidemiology and assay performance, is essential. The protocol should include (1) a standardized six-month temporary deferral for grey zone or low-level reactive donors; (2) mandatory follow-up testing using both serologic and NAT assays; and (3) conditional re-entry for donors with non-reactive follow-up testing results across all TTIs markers. Integration of this protocol into national hemovigilance and donor management systems would promote an evidence-based approach to donor eligibility, preserving safety while enhancing donor pool sustainability.

## 5. Study Limitations

This study was conducted at a single tertiary hospital blood bank in Saudi Arabia, which may limit the generalizability of the findings to other regions or donor populations with differing demographic or epidemiological characteristics. Although we analyzed follow-up serologic patterns, confirmatory diagnostic testing, such as immunoblot, was not performed, limiting our ability to determine the true infection status of grey zone or reactive results. Moreover, most donors did not return for subsequent donations, and the low donor retention rate observed in this study limited the assessment of longer-term seroconversion outcomes or follow-up trends.

## 6. Conclusions

This study provides one of the first large-scale evaluations of reactive and grey zone serologic screening results among blood donors in Saudi Arabia, examining over 38,000 donations across six mandatory TTIs markers. Our findings highlight the need for structured protocols for managing grey zone results, particularly those involving HBV markers such as anti-HBc, which may reflect early or occult infection rather than assay variability alone. Although a few grey zone donors seroconverted upon follow-up, these cases highlight the need for careful consideration of grey zone reactivity rather than complete dismissal.

At the same time, our data reveal that only a small number of grey zone donors returned for subsequent donation, suggesting a potential loss of donors. This may be due to unclear communication, absence of structured follow-up procedures, and donor perceptions of ineligibility, which has a direct impact on blood supply sustainability. We introduced a structured donor re-entry protocol aligned with international guidelines, incorporating temporary deferral, confirmatory testing, and clear criteria for reinstatement. Such a protocol balances transfusion safety with donor retention and can guide national policy toward evidence-based management of equivocal serologic results. Adoption of this approach can reduce unnecessary donor loss while maintaining the integrity and safety of the blood supply.

## Figures and Tables

**Table 1:** Summary of serologic screening results for transfusion-transmitted infectious markers among 48,241 blood donors.

TTIs Marker	Number of Reactions (%)	Reactive on Repeat (%)	Non-Reactive on Repeat (%)
Anti-HBc	Non-reactive = 45,844 (95.0)	NA	NA
	Reactive = 2312 (4.8)	2254 (97.5)	57 (2.5)
	Grey zone = 76 (0.16)	24 (31.6)	52 (68.4)
HBsAg	Non-reactive = 45,918 (95.2)	NA	NA
	Reactive = 2292 (4.8)	2233 (97.4)	59 (2.6)
	Grey zone = 28 (0.06)	8 (28.6)	20 (71.4)
HCV	Non-reactive = 48,055 (99.6)	NA	NA
	Reactive = 147 (0.3)	116 (79)	31 (21)
	Grey zone = 39 (0.08)	11 (28)	28 (72)
HIV	Non-reactive = 48,124 (99.8)	NA	NA
	Reactive = 103 (0.2)	21 (20)	82 (80)
	Grey zone = 14 (0.03)	0 (0)	14 (100)
HTLV	Non-reactive = 48,126 (99.8)	NA	NA
	Reactive = 95 (0.2)	68 (71.58)	27 (28.4)
	Grey zone = 20 (0.04)	3 (15)	17 (85)
Syphilis	Non-reactive = 47,987 (99.5)	NA	NA
	Reactive = 218 (0.5)	201 (92.2)	17 (7.80)
	Grey zone = 36 (0.07)	11 (30.6)	25 (69.4)

NA; not applicable.

**Table 2:** Follow-up outcomes of blood donors who initially tested in the grey zone for TTIs, highlighting return rates and subsequent serologic reactivity.

TTIs Marker	Grey Zone Results	Returned Donors (%)	Reactive on Return (%)
**Anti-HBc**	76	20 (26)	3 (15)
**HBsAg**	28	5 (17.9)	0
**HCV**	39	7 (18)	0
**HIV**	14	2 (14.3)	0
**HTLV**	20	3 (15)	0
**Syphilis**	36	6 (16.7)	0

## Data Availability

Data are not publicly available due to the inclusion of sensitive donor information and privacy concerns. Access to the datasets analyzed during the current study may be considered upon reasonable request to the corresponding author, subject to institutional and ethical approval.
